# Anomalous Dentition

**Published:** 1841-12

**Authors:** 


*Anomalous Dentition.*—A young lady, Miss G. of this city, aged about sixteen years, called a few days since, to consult us concerning her teeth. She had in her upper jaw the two permanent central incisors, the two bicuspides on each side, the two permanent molares, and the temporary cuspidati, one on each side. The temporary lateral incisors had been extracted about three years before, and from the thinness of the alveolar ridge, from where they were taken, will probably never be replaced with permanent ones. The temporary cuspidati are quite loose, and have been so, as we were informed, for nearly two years, and from the examination which we made, we are inclined to believe that they will never be succeeded by others. Her lower temporary teeth were all shed at the proper time, and replaced by others.

We mention this anomalous case, not because similar ones have not occurred, for they are occasionally though rarely meet with, and some five or six have fallen under our own immediate observation, but as showing that nature in the production of these organs, as well as in many of her other operations, does sometimes depart from her established course, and as one, which, though of no practical value, must nevertheless be interesting to the physiologist. *Balt. Ed.*

